# Dialectical Behaviour Therapy for the Treatment of Emotion Dysregulation and Trauma Symptoms in Self-Injurious and Suicidal Adolescent Females: A Pilot Programme within a Community-Based Child and Adolescent Mental Health Service

**DOI:** 10.1155/2013/145219

**Published:** 2013-06-12

**Authors:** Keren Geddes, Suzanne Dziurawiec, Christopher William Lee

**Affiliations:** ^1^Rockingham Kwinana Child and Adolescent Mental Health Service, P.O. Box 288, Rockingham, WA 6968, Australia; ^2^School of Psychology and Exercise Science, Murdoch University, Murdoch, WA 6150, Australia

## Abstract

*Background*. The literature suggests a link between childhood trauma and maladaptive emotion regulation strategies, including nonsuicidal self-injury (NSSI) and suicidality. We assessed the impact of a pilot dialectical behaviour therapy (DBT) programme on reducing trauma-related symptoms and improving emotional regulation, suicidality, and NSSI in adolescents. *Methods*. Six adolescents attending a community mental health service received 26 weeks of DBT, together with a parent. Independent assessors collected measures on each participant at baseline, posttreatment, and three-month followup. We implemented further improvements over past research with the use of adolescent-specific outcome measures as well as independent assessment of treatment integrity, noted as problematic in previous studies, using videotapes. *Results*. Firstly, adolescents reported a decrease in trauma-based symptoms, suicidality, and NSSI following participation in the DBT programme that was maintained at the three-month followup. Secondly, adolescents also reported improved emotion regulation immediately following treatment, and this was maintained, albeit more moderately, three months later. Given the burgeoning demand on mental health services, it is notable that five of the six adolescents were discharged from the service following the DBT intervention. *Conclusions*. The results of this pilot programme suggest that DBT has the potential to improve the symptoms of this at-risk population.

## 1. Introduction

A large percentage of adolescents present at community-based mental health clinics following acts of nonsuicidal self-injury, such as cutting or burning, due to significant difficulties with self-regulation of their emotions [1–4]. These adolescents often report using self-injury strategies to overcome emotional numbing [3], and many experience ongoing suicidal ideation, while some go on to make at least one and often more suicide attempts [3, 5, 6]. Given the nature of their presenting difficulties, many would argue that these adolescents have an “emerging borderline personality structure” [7–10].

These distressed adolescents, and the family systems in which they have been developed, have been shown to be remarkably difficult to treat [11], so that many will graduate from child and adolescent mental health settings to become long-term patients of adult mental health services, with multiple hospital admissions due to high levels of dysfunction, extreme management issues, and treatment resistance [3, 12]. The challenges to government-funded health services, both in the public and private sectors within Australia and, indeed, internationally, are compelling [13]. 

There is a well-established body of literature linking NSSI and suicidal behaviours with emotional dysregulation [3, 14–16] and childhood traumatic experiences, such as physical and sexual abuse [17]. In fact, it has been argued that these behaviours are used as a compensation strategy in posttrauma adaptation, functioning to assist with intra- and interpersonal regulations [17]. Thus, emotion dysregulation and childhood trauma are argued to be intimately linked aspects of the developmental process underpinning NSSI and suicidal behaviours. Support for the primacy of emotional dysregulation as a mediator of self-injury was provided in a randomised controlled trial of cognitive behavioural therapy (CBT) for 15- to 35-year-olds presenting with these difficulties [18]. Results indicated that emotion regulation difficulties, specifically, impulse control and goal-directed behaviours, partially mediated significant reductions in self-injury; however, in contrast, measures of depression, anxiety, and suicidal thoughts played no mediating role. It was recommended, therefore, that interventions aimed at reducing self-injury need to specifically target emotional dysregulation, in preference to other associated mental health disorders.

A promising intervention aimed at improving emotional regulation is dialectical behaviour therapy (DBT), developed by Marsha Linehan [14, 19] to treat chronically suicidal women diagnosed with borderline personality disorder (BPD). Linehan's biosocial theory, central to this intervention, argues that BPD is principally the result of a dysfunctional emotion regulation system associated with instability of thoughts, emotions, behaviours, relationships, and self-image.

The original programme [20] was conducted over a one-year period, was highly structured, and included four specific treatment components: weekly individual psychotherapy; weekly group skills-based training; telephone consultation between sessions; and weekly team consultation-supervision meetings. Using principles and strategies drawn from behaviour therapy and Zen Buddhism, DBT is recognised as the first empirically validated treatment developed for adults with BPD [21] and has been accepted as an efficacious way of treating various populations experiencing emotional dysregulation difficulties [22, 23].

Research to date suggests that adult women diagnosed with BPD show improvements following DBT intervention. Specifically, DBT has been found to be effective in reducing targeted problem behaviours, such as self-injury and suicidality, thereby reducing hospital admissions and reducing treatment dropout rates in severely impaired populations. In less severe populations, DBT also appears to produce specific improvements in suicidal ideation, depression, and hopelessness [20, 23, 24].

In 1997, the adult DBT programme was modified (DBT-A) to suit 13- to 19-year-old suicidal adolescents presenting with borderline personality traits [25]. Treatment was reduced from one year to 12 weeks to enhance completion, and weekly individual psychotherapy was also provided, with family members included when family issues predominated. A family member was also included within the skills-training group to act as a coach, improve generalisation of treatment effects, and reduce family dysfunction. The number of skills taught was reduced and the language was simplified to improve learning within 12 weeks. A fifth skills module, “Walking the Middle Path,” was also added. Adolescents who completed the programme were also offered a 12-week follow-up patient consultation group, which relied on peer teaching and reinforcement, so that adolescents were able to help each other strengthen the skills learnt in the first three months of the programme.

At the time of the current study development, only three clinical trials of the DBT-A group had been conducted on adolescents presenting with suicidal and self-injury behaviours. The first was a nonrandomised controlled trial for adolescents, aged 14–19 years, who were predominantly females, and compared a DBT group with a treatment-as-usual group [26]. The DBT-A group included adolescents who had attempted suicide and who also presented with a minimum of three additional borderline features. The treatment-as-usual group included suicide attempters only. No difference was found between the groups on rates of suicide attempts; however, DBT-A adolescents had a lower rate of treatment dropout and fewer days of inpatient care. Within the DBT-A group, significant reductions in suicidal ideation, anxiety, and depression were shown. Significant reductions were also found in self-reported BPD symptoms in the areas of confusion about self, impulsivity, emotional dysregulation, and interpersonal problems [26].

Another nonrandomised controlled trial was conducted with adolescent girls residing in three juvenile rehabilitation units [27]. This four-week adaptation of DBT found mixed results when comparing measures of behavioural problems and staff punitive responsiveness between groups of adolescents receiving DBT in a mental health unit with those receiving DBT in a general population unit, against a treatment-as-usual group. Notably, no inclusion or exclusion criteria were used and adolescents in the two DBT comparison groups were distinctly different from each other in terms of behavioural problems, with the mental health adolescent group presenting with more severe mood and thought disturbance. Not unexpectedly, the mental health group showed a marked reduction in problem behaviours following the four-week DBT intervention; however, the general population group showed no reduction in problem behaviours.

In the third trial, adolescents aged between 14 and 17 years were treated with a two-week adaptation of the original DBT-A programme [25] in an inpatient psychiatric unit [28]. Comparisons were made with a treatment-as-usual group and indicated similar improvements in depression, suicidal ideation, and hopelessness for both groups. However, the DBT-A group also showed significantly reduced behavioural incidents on the ward.

A review of the above studies highlighted that the quality of the data was highly questionable due to factors of selection bias, confounding variables, difficulties with outcome measures, and measurement errors [29]. The review concluded that the efficacy of DBT-A in reducing mental health symptoms in adolescents was yet to be established. Specific recommendations for future research were made: firstly, that treatment needed to occur in outpatient settings to minimise the influence of confounding environmental factors on treatment outcome, as can occur in hospital-based settings; and secondly, that more developmentally appropriate measures be used, as all studies incorporated adult and/or child measures that were likely to have been clinically insensitive to the symptomatology of adolescent presentations.

Apart from these three trials, there has also been a study using a within-case design to test DBT with adolescent females, aged between 13 and 19 years, presenting with nonsuicidal self-injurious, and suicidal behaviour within an outpatient setting [30]. The programme consisted of weekly individual therapy, a weekly multifamily skills group, and telephone support, conducted over a 16–24-week period. Results from this study were promising, with adolescents showing reductions in nonsuicidal, self-injurious, and suicidal behaviour, as well as improvements in interpersonal relationships, identity disturbance, impulsivity, and depression over the course of the treatment and at one-year followup. However, given the lack of a supervision/consultation group, it could be argued that treatment integrity was somewhat problematic, given this was one of the four treatment components specified in the original model [25]. More recently, it has also been noted that without treatment adherence ratings, it is difficult to assess whether or not patients actually receive DBT-A treatment [21]. In this regard, it is noteworthy that the most recent study to date [30] did not address the issue of treatment adherence.

The aim of the present study was to develop and pilot a DBT-A programme, based on the original adolescent programme [25], and assess its feasibility and efficacy in treating a community-based outpatient population of adolescents presenting with NSSI and suicidality. Based on the literature, two specific research questions were addressed: (1) does DBT-A lead to improvements in adolescents' capacity to regulate their emotions? and, given the links between emotion dysregulation, borderline personality symptoms, and early trauma experiences, (2) would improvements in emotion regulation produce comparable reductions in trauma-related symptoms, self-injurious behaviours, and suicidality? From the outset, attention was paid to ensure that the measures were appropriate to the age group. In addition, all measures were collected by independent assessors to evaluate outcome.

Importantly, emotional dysregulation was operationalized within the present study as the “fear of losing control over one's emotions or of one's behavioural reactions to emotions” [31, page 241] and relates to the measure of emotional regulation developed for the current study, the Modified Affective Control Scale for Adolescents (MACS-A) [32]. In the development of the original adult measure, the Affective Control Scale (ACS) [31], the fear of fear concept was extended to include the fear of other strong emotions, specifically, depression, positive emotion, and anger, with the focus on attention to internal events, and the perceived ability of individuals to cope with strong emotions. Reflection on the original work of Linehan [14] reveals her acknowledgement of the part played by the fear of anger and of losing control over anger in self-injuring borderline patients, stating “In almost all cases, the under expressive borderline individuals have a marked fear and anxiety about anger expression; at times they fear that they will lose control if they express even the slightest anger, and at other times they fear that targets of even minor anger expression will retaliate” (page 16). Indeed, research in adult populations has found that the fear of one's own emotions is associated with maladaptive psychological outcomes, such as posttraumatic stress disorder, generalized anxiety disorder, and symptoms of borderline personality disorder [33–35].

Two predictions were generated within the present study. First, it was predicted that 14- to 16-year-old adolescents presenting at a community-based Child and Adolescent Mental Health Service with NSSI and suicidality would report a decrease in trauma-based symptoms on the Trauma Symptom Checklist for Children [36] and reduced acts of self-injury and suicidal thoughts, following participation in a 26-week DBT-A programme. Second, it was predicted that a reduction in trauma symptoms, self-injury and suicidal thoughts would be associated with improvements in emotion regulation as measured by a decrease in scores on the Modified Affective Control Scale for Adolescents (MACS-A).

## 2. Method

### 2.1. Ethical Considerations

This study received joint approval from the Human Research Ethics Committee at Murdoch University, Perth, WA, Australia, and the Human Research Ethics Committee of the South Metropolitan Area Health Service, WA, Australia. All adolescents and their parents participating in this research did so voluntarily.

### 2.2. Participants

Six female adolescents aged between 14.6 years and 15.7 years, with a mean age of 15.1 years, participated in this pilot programme. Three adolescents were current clients of the Child and Adolescent Mental Health Service (CAMHS), while the remaining three adolescents had been recently referred to CAMHS. A parent accompanied all adolescents to the family skills-training component, so that the final group included four mothers and two fathers. Of the six dyads participating, four dyads completed the entire 26-week programme and the remaining two dyads were withdrawn from the programme by their parents, three weeks prior to treatment completion. All six dyads were available for posttreatment (t2) assessments, while five dyads were available for three-month followup (t3).

Adolescents were considered appropriate for the DBT-A programme based on the following criteria:

 
*Inclusion Criteria*
  - Aged between 13 and 18 years.
  - Average cognitive ability (clinician's notes, school records) and established reading level (year 5), as measured by the Neale Analysis of Reading Ability [37].
  - Referred to the service because of deliberate self-harm and/or suicidal ideation in the previous 12 months.
  - A minimum of three BPD features, as determined by clinician assessment and according to DSM-IV criteria.
 
*Exclusion Criteria*
  - A primary diagnosis of a psychotic disorder.
  - A primary diagnosis of substance abuse.
  - An intellectual disability.

### 2.3. Design

The process of implementation and assessment of this 26-week pilot group was as follows:

- *8-week engagement and commitment to DBT-A:* treatment contracts signed by adolescents and parents, and individual and group clinicians. (t1) Pretreatment measures administered prior to engagement.
- *18-week DBT-A treatment: *(t2) posttreatment measures administered at completion of programme.
- *Followup: *(t3) 3-month follow-up measures administered.

### 2.4. Measures

#### 2.4.1. Intake Measures

*Assessment of Borderline Personality Features.* Past researchers [25] investigating the effectiveness of DBT-A in the treatment of suicidal adolescents presenting with borderline features have used a structured clinical interview, the SCID-11 [38], to determine suitability for the DBT-A programme. However, the SCID-11 was designed for use with individuals aged 18 years and older and its construct validity and predictive ability with adolescents has been challenged [7]. Therefore, assessment of borderline features for the purposes of this study was determined in a clinical interview, by reference to criteria set out in the Diagnostic and Statistical Manual IV (1994).

*Neale Analysis of Reading Ability [37].* Adolescents were screened individually on this measure of reading ability. The Neale Analysis is a standardised measure of both reading accuracy (word recognition in context) and reading comprehension (assessed by the ability to answer a series of questions about a passage). A reading age equivalent of age 10 was needed to understand programme content.

#### 2.4.2. Outcome Measures

*Self-Harm/Suicidal Thoughts Questionnaire: Parent and Adolescent Versions.* A self-report questionnaire, developed specifically for this programme, consisted of three sections: section one assessed various forms of self-injurious behaviours, inclusive of abuse of medications, burning, scratching or cutting, and hitting or punching the self; section two assessed the extent of each self-injurious behaviour, including age when commenced, frequency, and seriousness (requiring medical treatment); section three assessed frequency of suicidality.

*Modified Affective Control Scale for Adolescents (MACS-A) [32].* This 41-item self-report questionnaire was developed specifically for the current programme as a measure of adolescent's capacity to regulate their emotions and is a reworded version of an adult measure of emotion regulation, the Affective Control Scale (ACS) [39]. This scale consists of four subscales that measure fear of anger (8 items), fear of depression (8 items), fear of anxiety (13 items), and fear of positive emotion (12 items). Participants rate each item on a 7-point Likert scale, from “very strongly disagree” to “very strongly agree,” with a neutral midpoint. Individual subscale scores are computed as the mean of the total number of items contained in the subscale. An overall scale score is computed as the mean of all 41 responses, with the higher the mean score, the higher the perceived fear of emotion/s and the greater the difficulty in emotional regulation. The MACS-A was found to be internally consistent in both clinic and nonclinic adolescent samples and to effectively discriminate between these two groups, with the exception of the fear of positive emotion subscale [32]. For this reason, results from this subscale were not included in the current analysis.

*Trauma Symptom Checklist for Children (TSCC) [36].* This 54-item self-report measure assesses a variety of symptoms associated with trauma experiences in children, aged between 8 and 16 years. Participants rate each item on a 5-point Likert scale, from “not at all characteristic” to “very characteristic” of themselves. The measure has two validity scales (underresponding and overresponding) and six clinical scales: anxiety, depression, anger, posttraumatic stress, sexual concerns and dissociation. There are also two additional subscales: sexual concerns (sexual preoccupation and sexual distress) and dissociation (overt dissociation, fantasy). The reliability and validity of the TSCC were independently examined in a clinical sample of adolescents, including a percentage with a history of sexual abuse [40]. These researchers found that all six scales and four subscales of the TSCC were a reliable and valid measure of distress in a psychiatric adolescent sample. The current study does not report on results from dissociation (fantasy) or the sexual concerns subscales.

## 3. Procedure

Both parent/s and adolescent attended an initial appointment with the programme coordinator. Programme content and commitments were explained, and consent forms were completed including consent for the videotaping of all individual and group sessions.

### 3.1. DBT-A Programme Development (Life Surfing)

This programme was developed over the course of 2005 and 2006, based on a previous adaptation of DBT for adolescents [25] that was modified from adult programme content [14, 19].

Similar to previous versions [25], there were four treatment components: individual therapy, a multifamily skills-training group, phone consultation, and a therapist supervision/consultation group. All four components were built into this pilot programme, entitled “Life-Surfing,” with the exception of the out-of-hours phone consultation, due to lack of clinician indemnity. In addition, the family skills group ran for 18 weeks, whereas the original DBT-A programme [25] ran for 12 weeks. The following is a description of the components of the DBT-A, “Life-Surfing” programme.

*(1) Individual Therapy.* Adolescents were seen weekly (twice weekly, if needed) for the length of the programme. The structure of individual treatment was in line with the standard DBT protocol [14], which set out a prescriptive treatment hierarchy consisting of four stages:

- a pretreatment stage involved orienting the client to treatment, gaining commitment, and agreeing on the goals of treatment;
- first stage focused on client stability, connection, and safety and structured with a specific subhierarchy of therapeutic goals:
  - decrease life-threatening behaviours;
  - decrease therapy-interfering behaviours;
  - decrease quality-of-life interfering behaviours;
  - increase behavioural skills;
- second stage involved exposure and emotional processing of the past;
- third stage looked at increasing respect for the self- and individual goals.

As recommended [25], family members were invited to join individual sessions when systemic issues dominated.

*(2) Family Skills Training Group.* This group was highly structured and ran for two hours each week. Sessions were psychoeducational in focus with an emphasis on acquisition and practicing of new skills. There were five modules written for the DBT-A programme: Core Mindfulness, Distress Tolerance, Emotion Regulation, Interpersonal Skills, and Middle Path. Modules were presented in the above sequence, and each module ran for four weeks, with the exception of the core mindfulness module, which ran for two weeks. Core mindfulness skills were revisited throughout the length of the programme, and, by the ninth week of the skills training group, adolescents and/or parents volunteered to run the mindfulness exercise that commenced each week's group skills-training session. The family skills-training group ran for 18 weeks.

*(3) Phone Consultation.* Provided during business hours and focused on helping adolescents use skills learnt during the programme.

*(4) Supervision/Consultation Team.* A group of interested CAMHS clinicians formed the supervision/consultation team. This team met every week for two hours during the initial stages of the programme development, and then throughout the length of the programme implementation. This was a multidisciplinary team consisting of individuals trained in clinical psychology, social work, and psychiatry. Initially, the purpose of this team was to develop the programme content and structure, including programme viability, funding, ethical considerations, and clinical concerns, as well as to provide ongoing education regarding the implementation of the DBT model. Once treatment commenced, this group provided clinical supervision and addressed treatment integrity through the review of videotaped individual and group skills-training sessions, as recommended [14].

### 3.2. Programme**  **Commitments

Adolescents, parents, individual and group skills clinicians, and supervision/consultation team members made formal commitments to the DBT-A programme prior to its commencement. These commitments formed the basis of the programme contracts signed by adolescents, parents, and clinicians as follows:

- Adolescents agreed to
  - weekly individual therapy for the length of the programme (26 weeks);
  - weekly family skills-training group (18 weeks);
  - videotaping of all individual and group sessions.
- Parents agreed to
  - weekly family skills-training group (18 weeks);
  - videotaping of all group sessions.
- Clinicians agreed to work with clients for 6 months which included
  - weekly individual therapy: 26 weeks (individual therapists);
  - weekly family skills group: 18 weeks (group therapists);
  - clinical consultation/supervision group (2 hrs/week);
  - videotaping of all therapy sessions.

Individual and group clinicians also agreed to take no more than two weeks leave within the six-month period of the programme.

### 3.3. Clinician Experience and Training

All members of the team received one full day of in-house DBT training, conducted by two clinical psychologists who were members of the DBT consultation/supervision team. One of these clinicians had attended a five-day intensive training workshop on DBT conducted by Linehan's training organization, Behavior Tech., while the other clinician was at that time coordinating an adult DBT programme and was also a coleader of the group skills-training component of that programme.

### 3.4. Treatment Adherence Check

An estimate of individual and group therapist adherence to the basic strategies of DBT was conducted by rating a random sample of five recorded individual therapy sessions and five recorded group skills-training sessions against a DBT adherence rating scale. The third author, who is a recognised trainer in DBT, rated these sessions. The mean adherence rating was 4.0 out of a possible 5.0, with a range of between 3.5 and 4.5.

## 4. Results

The effect of the programme was investigated by using group mean scores to calculate the Wilcoxon signed-rank test at t1 (pretreatment), t2 (posttreatment), and t3 (three-month followup), on the TSCC and MACS-A. Effect size (*r*) was also calculated, based on the standardised difference between two means for dependent groups. Initial interpretation of effect size (*r*), set out below, used the general convention provided by Cohen [41] as  follows:  *r* : small > 0.10, medium > 0.3, large > 0.5. However, in interpreting the meaningfulness of effect sizes, it has been noted that a practically significant result is one that has meaning in the real world, and interpretation is a complex and subjective process [42].

### 4.1. Changes in Suicidal Thoughts and Deliberate Self-Harm Behaviours

Prior to commencement of therapy, all six adolescents were reporting suicidal thoughts at a minimum of twice weekly to a maximum of several times a day. One adolescent had also attempted suicide on more than one occasion. During treatment, and 12 months after treatment, no adolescent attempted suicide. At the end of the programme, one adolescent reported continuing suicidal thoughts once a week, one other adolescent reported suicidal thoughts once a month, while the remaining four adolescents reported no further suicidal ideation.

All adolescents reported regular DSH for at least three months prior to entry to DBT-A treatment. At the end of the programme, five of the six adolescents had ceased to self-harm over the course of treatment, while the remaining adolescent reported a 50% reduction in DSH events.

### 4.2. Comparison between Trauma-Related Symptoms at Pretreatment (t1), Posttreatment (t2), and 3-Month Followup (t3)

All participants produced valid TSCC protocols at t1, t2, and t3, as indicated by nonsignificant levels of over- and underresponding.  Table 1 presents pre- and posttreatment comparisons on subscale scores of the TSCC, revealing large and statistically significant decreases in mean scores on self-reported anxiety (*z* = −2.07, *P* < 0.05, *r* = 0.60), depression (*z* = −2.03, *P* < 0.05, *r* = 0.59), anger (*z* = −2.0, *P* < 0.05, *r* = 0.58), and posttraumatic stress (*z* = −2.02, *P* < 0.05, *r* = 0.58).

Table 2 presents pretreatment (t1) and three-month (t3) followup comparisons on subscale scores of the TSCC, revealing large and significant decreases in mean scores on self-reported anxiety (*z* = −2.02, *P* < 0.05, *r* = 0.64), depression (*z* = −2.02, *P* < 0.05, *r* = 0.64), and posttraumatic stress (*z* = −2.02, *P* < 0.05, *r* = 0.64). There was also a large decrease in mean scores at three-month followup on self-reported anger; however, this decrease was nonsignificant (*z* = −1.83, *P* < 0.05, *r* = 0.58).

### 4.3. Comparison between Emotional Regulation at Pretreatment (t1), Posttreatment (t2), and 3-Month Followup (t3)

Table 3 presents pre- and posttreatment comparisons on the whole-scale and subscale scores of the MACS-A. On the whole-scale measure of fear of emotion, there was a large but statistically nonsignificant decrease in mean scores (*z* = −1.78, *P* > 0.05, *r* = −0.51) from pretreatment to posttreatment. Comparison of subscale mean scores revealed a large and statistically significant decrease in self-reported fear of anger between pretreatment and posttreatment (*z* = −2.20, *P* < 0.05, *r* = −0.64) and a small but nonsignificant decrease in fear of depression (*z* = −0.95, *r* = −0.28); however, the decrease in fear of anxiety was both negligible and nonsignificant (*z* = −0.21, *P* > 0.05, *r* = −0.06).

Table 4 presents pretreatment (t1) and three-month follow-up (t3) comparisons on the whole-scale and subscale scores of the MACS-A. Results from the whole-scale measure of fear of emotion indicate a moderate decrease in mean scores from pretreatment to three-month posttreatment; however, this change was not statistically significant (*z* = −1.21, *P* > 0.05, *r* = −0.38). With regard to the subscales, results reveal a large and statistically significant decrease in self-reported fear of depression (*z* = −1.21, *P* > 0.05, *r* = −0.64) three months after treatment. There was also a moderate but nonsignificant decrease in self-reported fear of anger (*z* = −0.1.48, *P* > 0.05, *r* = −0.47) and fear of anxiety (*z* = −1.21, *P* > 0.05, *z* = −0.38) three months after treatment.

## 5. Discussion

The overarching goal of the current study was to develop and pilot a DBT-A programme, based on the work of Miller et al. [25], and to assess both its feasibility and efficacy in a community-based population of adolescent females presenting with DSH and suicidal thoughts. A further focus was to assess the central tenet of Linehan's [14] biosocial theory, which argues that emotional dysregulation underpins the DSH and suicidal behaviours associated with a borderline personality.

In discussing the results of the present study, recognition is given to recent requests for scientifically valid and practical research [43]. When interpreting the practical significance of findings, the majority of studies reporting on effect sizes fail to interpret them in meaningful ways, with three important factors being highlighted when considering interpretation [42, page 34]:

1. context: small effects may be linked to large consequences and that small effects may be cumulative;
2. contribution to knowledge when conducted in real-world settings;
3. Cohen's criteria.

Certainly, when looking at the practical significance of the current findings, it is important to emphasise the difficulties of engaging with and treating this population of adolescents. Effective early interventions are needed, given the high costs of multiple hospital admissions and the likely probability of continued treatment through to adulthood. Given this, it could be argued that even small effect sizes, whereby one or two adolescents respond well to treatment, can be considered practically significant over a longer term.

Results of the current study provided support for our initial hypothesis that adolescents participating in the DBT-A programme would report a decrease in trauma-based symptoms on the TSCC and reduced acts of self-injury and suicidal thoughts. Adolescents reported large and significant reductions in their symptoms of anxiety, anger, depression, and posttraumatic stress immediately following DBT-A treatment. Three months following the end of treatment, adolescents continued to report significantly large reductions in anxiety, depression, and posttraumatic stress symptoms and a large reduction in anger symptoms. While there was no meaningful reduction in dissociative symptoms immediately following treatment, moderate reductions were reported three months after treatment completion. These results are consistent with earlier research where significant reductions in anger, depression, and dissociative symptoms, as measured by the TSCC, were found in a group of self-harming and suicidal adolescents following DBT-A treatment at a community outpatient clinic [44]. Furthermore, the present study findings regarding the cessation of self-harm and reduction in suicidal thoughts by the end of the programme are consistent with earlier DBT-A research [45].

Results from the present study also provided support for our second hypothesis that adolescents participating in the DBT-A programme would show an improved capacity to regulate their emotions, as measured by the MACS-A. Importantly, on average, adolescents reported a large reduction in their fear of emotion following treatment, and this decrease was maintained, albeit more moderately, three months following treatment. On the fear of anger subscale scores of the MACS-A, adolescents reported a large a decrease following treatment, but this was not maintained three months after treatment. On average, adolescents also reported a small reduction in their fear of depression immediately following treatment, and, encouragingly, this reduction became both large and significant three months after treatment completion. Although there were no reported meaningful improvements in adolescents' fear of anxiety on completion of treatment, moderate reductions were reported three months later.

Overall, these results suggest that this group of adolescents reported an improved capacity to manage their strong emotions following participation in the DBT-A programme, and that these gains were maintained in varying degrees three months later. In particular, they reported that their fear of depression and fear of anxiety continued to decrease over the three-month period following the end of all treatments. This result supports previous findings [46] that treatment gains made by self-harming adolescent females following DBT intervention were not only maintained six months after treatment, but continued to show further improvement. Given the importance of the therapy relationship as a container of emotion, particularly in this population [47], the finding that adolescents participating in the current programme were able to report continuing reductions in their fear of depression and anxiety three months after the cessation of all treatment is encouraging.

Importantly, specific recommendations made in a review [29] of the data from past clinical trials were addressed within the current study. First, treatment occurred within a community-based outpatient setting, reducing the confounding effects of environmental factors that are likely to occur within an inpatient setting. Second, the current study used developmentally appropriate measures of outcome, including the development of a measure that specifically assessed emotional regulation in adolescents in the form of perceived fear of emotions, shown by past studies to be linked to problematic mental health outcomes, including borderline personality symptoms. Finally, attention was paid to treatment fidelity. The present study determined an acceptable level of DBT treatment adherence from rating a random sample of taped individual and group sessions.

Confidence in the findings from the present study was also increased by the use of an independent researcher to collect pre-, post-, and follow-up measures, thereby minimising the potential for bias that has occurred in other studies [30]. Furthermore, determining adolescents' capacity to understand the programme material through formal assessment of their reading level and comprehension has not occurred in previous studies and adds to the reliability of the current results.

From a service provision perspective, this study contains some other specific strengths. Of the six adolescents participating in the programme, only one remained in therapy following the end of the programme. Notably, one other adolescent, who had been treated at the clinic at various times since she was six years of age, did not return for any further treatment following the ending of the DBT-A programme, with followups revealing that she had moved into full-time work and training. Given that long waiting lists for admission to CAMHS clinics is a common problem, combined with the high probability for these adolescents to move into adult mental health services, this finding is of great practical significance.

The current study also had some limitations inherent within its design. Specifically, as a pilot study, it lacked a control group, and, therefore, specific conclusions about the effectiveness of the programme cannot be made. Neither can it be concluded that the DBT-A programme was more effective than treatment-as-usual. However, it can be said that four of the six adolescents completed the programme, which is comparable to the 62% completion rate of other studies [45]. Given the high rates of treatment dropout usual for this population, this finding provides some support for the potential effectiveness of DBT-A with these adolescents. A further limitation of this study was the potential influence of demand characteristics on self-report measures. Future studies would benefit from the collection of more objective markers of treatment effectiveness, for instance, collateral information from parents and schools.

## 6. Conclusions

In this pilot programme, we found preliminary evidence that DBT-A for the treatment of NSSI and suicidality in adolescents was both feasible and efficacious. Despite limited funding for this project, a DBT-A programme was successfully developed and piloted in the community setting in which these high-risk adolescents are most likely to seek initial treatment. Furthermore, results suggest that emotional regulation and trauma symptoms are important constructs to be monitored over time.

## Figures and Tables

**Table 1:** Pre- (t1) and posttreatment (t2) comparisons on the TSCC for the 6 participants.

	Pretreatment (t1)		Posttreatment (t2)		Statistics (t1 to t2)	
TSCC	Mean	SD	Mean	SD	Wilcoxon's test	Effect size *r*
Anxiety	58	9.01	48.5	6.25	*P* = 0.046*	0.6
Depression	64	9.21	58.33	9.70	*P* = 0.038*	0.59
Anger	68	11.63	58.17	8.66	*P* = 0.042*	0.58
PTS	60	10.71	53.83	10.61	*P* = 0.043*	0.58
Dissociation	63	16.9	61.17	18.43	*P* = 0.92	0.03

*sig < 0.05 (2 tailed).

**Table 2:** Pre- (t1) and three-month follow-up (t3) comparisons on the TSCC (*n* = 5).

	Pretreatment (t1)		3-month followup (t3)		Statistics (t1–t3)	
TSCC	Mean	SD	Mean	SD	Wilcoxon's test	Effect size *r*
Anxiety	67	5.4	49	9.06	*P* = 0.043*	0.64
Depression	71	10.07	51.4	11.78	*P* = 0.043*	0.64
Anger	60	8.46	48	6.44	*P* = 0.068	0.58
PTS	64	6.52	49.6	12.34	*P* = 0.043*	0.64
Dissociation	68	13.01	54.6	9.5	*P* = 0.138	0.47

*sig < 0.05 (2 tailed).

**Table 3:** Pre- (t1) and posttreatment (t2) comparisons on the MACS-A (*n* = 6).

	Pretreatment (t1)		Posttreatment (t2)		Statistics (t1 to t2)	
MACS-A	Mean	SD	Mean	SD	Wilcoxon's test	Effect size *r*
Fear of Anxiety	4.57	1.27	3.32	1.22	*P* = 0.833	−0.06
Fear of Anger	3.86	0.69	3.73	0.45	*P* = 0.028*	−0.64
Fear of Depression	4.07	1.24	3.33	0.91	*P* = 0.34	−0.28
Fear of Emotion	4.00	0.83	**3.40**	0.55	*P* = 0.075	−0.51

*sig < 0.05 (2 tailed).

**Table 4:** Pre- (t1) and three-month follow-up (t3) comparisons on the MACS-A (*n* = 5).

	Pretreatment (t1)		3-month followup (t3)		Statistics (t1 to t3)	
MACS-A	Mean	SD	Mean	SD	Wilcoxon's test	Effect size *r*
Fear of Anxiety	3.9	0.76	3	1.11	*P* = 0.225	−0.38
Fear of Anger	4.6	1.41	3.36	0.96	*P* = 0.138	−0.47
Fear of Depression	4.4	1.03	2.88	0.73	*P* = 0.042*	−0.64
Fear of Emotion	4.01	0.91	3.1	0.8	*P* = 0.225	−0.38

*sig < 0.05 (2 tailed).
